# Synergistic adaptation of rice root phosphorus uptake kinetics and leaf carbon–nitrogen metabolism under low-phosphorus conditions

**DOI:** 10.3389/fpls.2026.1836788

**Published:** 2026-05-18

**Authors:** Chenglong Guan, Fenglou Ling, Tai Ma, Ming Miao, Zexin Qi, Wenzheng Sun, Ye Wang, Mengru Zhang, Qiang Zhang, Zhian Zhang, Hongjun Wang

**Affiliations:** 1 Agronomy College, Jilin Agricultural University, Changchun, China; 2 Jilin Academy of Agricultural Sciences (Northeast Agricultural Research Center of China), Changchun, China; 3 Jilin Academy of Agricultural Sciences (Jilin Agricultural Products Quality Supervision and Inspection Center), Jilin, China

**Keywords:** leaf carbon and nitrogen metabolism, low phosphorus treatment, rice, root phosphorus uptake kinetics, synergistic adaptation mechanism

## Abstract

**Background and objective:**

Phosphorus (P) deficiency is a major constraint on rice growth and productivity. Clarifying the physiological basis of root–shoot coordination under phosphorus limitation is essential for improving phosphorus-use efficiency in rice.

**Methods:**

Following a preliminary phenotypic screening of 156 rice germplasm resources, four rice cultivars representing two contrasting low-P tolerance groups were selected, including two tolerant cultivars (J873 and J705) and two sensitive cultivars (T35 and L20). The physiological responses of these four cultivars to low-P stress were systematically compared using the Claassen–Barber nutrient depletion method, gas exchange measurements, and enzymatic assays.

**Results:**

Phosphorus deficiency was associated with marked changes in root phosphorus uptake kinetics. The tolerant cultivars developed an uptake pattern characterized by the coexistence of high uptake capacity (high *I*_max_) and high affinity (low *K*_m_), improving phosphorus acquisition efficiency. In leaves, these cultivars maintained higher stomatal conductance, supporting transpiration-driven nutrient transport, and exhibited greater acid phosphatase activity, consistent with enhanced intracellular phosphorus recycling. Along with delayed leaf senescence, these responses alleviated the biochemical limitations on photosynthesis, with the net photosynthetic rate at day 21 remaining 39.47–45.09% higher than that of the sensitive cultivars. Metabolic analysis indicated that enhanced sucrose phosphate synthase activity was accompanied by a 42.18–43.10% increase in the root-to-shoot ratio, reflecting greater carbon allocation to roots, while the accumulation of free amino acids contributed to the maintenance of carbon–nitrogen metabolic balance. Structural equation modeling indicated that under low-P conditions, biomass accumulation was jointly regulated by photosynthetic carbon assimilation and coordinated carbon–nitrogen metabolic processes.

**Conclusion:**

Coordinated regulation of root phosphorus uptake kinetics and SPS-mediated carbon partitioning is a key physiological strategy underlying rice adaptation to low-P conditions.

## Introduction

Phosphorus (P) is indispensable for plant growth and development because it underpins multiple core physiological functions, including energy transfer, photosynthesis, and carbohydrate metabolism (Wang et al., 2025). Despite the substantial amount of total P present in most soils, only a limited fraction remains bioavailable, as the majority is rapidly adsorbed or immobilized by soil minerals (Luo et al., 2024). This constraint is particularly critical for rice (*Oryza sativa* L.), a globally important staple crop, whose growth and yield are highly responsive to P supply. Under modern intensive farming systems, the sustained dependence on P fertilizer has not only increased production costs but has also intensified environmental problems such as aquatic eutrophication (Dissanayaka et al., 2018). Therefore, a clearer understanding of the physiological basis of low-P tolerance in rice and more effective exploitation of P-efficient germplasm are crucial for sustainable agricultural production.

Adaptation to normal phosphorus (NP) is not governed by a single trait but by the coordinated operation of multiple physiological processes, spanning external P acquisition by roots and internal P utilization within the plant (Jiang et al., 2025). As roots represent the primary interface for P uptake, their adaptive behavior has remained a major focus of low-P (LP) research. Studies have emphasized the morphological plasticity of root system architecture, particularly traits such as lateral root proliferation and root hair elongation, which enlarge the soil exploration domain (Mori et al., 2016; Wang et al., 2023). However, at the actual root–soil uptake interface, the kinetic properties of transmembrane phosphate (Pi) transport may exert a more direct regulatory influence on phosphorus acquisition (Huang and Zhang, 2020). In this context, the classical Michaelis–Menten parameters, namely the maximum uptake rate (*I*_max_) and affinity constant (*K*_m_), provide quantitative descriptors of the functional capacity of the root phosphate transport system (Epstein and Hagen, 1952). Theoretical analyses have suggested that genotypes with higher *I*_max_ may have superior nutrient-acquisition potential under LP conditions (Mori et al., 2016). Conversely, current screening systems for low-P tolerance in rice still depend mainly on macroscopic phenotypic traits, particularly root morphology. Consequently, systematic and quantitative evidence regarding genotypic variations in uptake kinetic parameters and their relative contributions to low-P tolerance remains insufficient (Singh et al., 2017; Bui et al., 2019; Schuster et al., 2024).

A large proportion of phosphorus absorbed by plants is directly involved in photophosphorylation and carbon assimilation. Consequently, LP commonly suppresses the net photosynthetic rate (*P*_n_) through the combined action of stomatal and non-stomatal limitations (Carstensen et al., 2018). Under prolonged or severe low-P stress, increasing evidence suggests that non-stomatal constraints associated with reduced mesophyll metabolic activity become the dominant source of photosynthetic inhibition (Pan et al., 2021; Shu et al., 2023). This metabolic restriction is closely linked to the regulation of key enzymes, including ribulose-1, 5-bisphosphate carboxylase/oxygenase (Rubisco), which supports carbon fixation, and sucrose phosphate synthase (SPS), which governs the export of photosynthates towards sink tissues (Makino et al., 1994; Bracher et al., 2017). Accordingly, low-P-tolerant cultivars should preserve efficient carbon assimilation under phosphorus limitation and sustain an effective source–sink transport system that ensures the timely allocation of photosynthates to roots for growth and nutrient uptake. Although root phosphorus uptake efficiency and shoot metabolic adjustment to phosphorus deficiency have both been widely investigated, their integration has rarely been quantitatively examined within a unified statistical framework in rice. This integrated perspective is essential for elucidating how root nutrient acquisition and shoot carbon–nitrogen metabolism act in concert to determine plant performance under phosphorus limitation.

Building on this perspective, this study tested the central hypothesis that low-P tolerance in rice is regulated by a synergistic regulation between root phosphorus uptake kinetics and leaf carbon–nitrogen metabolism. We propose that root uptake capacity primarily determines the initial acquisition of phosphorus. In contrast, the coordinated reallocation of carbon and nitrogen metabolites becomes increasingly important for maintaining plant performance under prolonged phosphorus limitation. To test this hypothesis, four rice cultivars with contrasting low-P tolerance levels were selected based on preliminary germplasm screening. A modified nutrient depletion approach combined with a hydroponic low-P cultivation system was employed to quantify root P uptake kinetic parameters and assess P acquisition capacity. In parallel, the temporal responses of leaf gas exchange, major carbon metabolism-related enzymes, and associated metabolites to low-P stress were systematically examined. This study was designed to address three objectives: (1) defining cultivar-dependent variation in root P uptake kinetics under contrasting tolerance backgrounds, (2) clarifying the response patterns of key enzymes and metabolites involved in leaf carbon–nitrogen metabolism under phosphorus deficiency, and (3) elucidating the synergistic association between root uptake efficiency and shoot metabolic adaptation through Random Forest analysis and Structural Equation Modeling (SEM). These results clarify the physiological basis of low-P tolerance in crops from the perspective of root–shoot coordination and provide a theoretical foundation for the genetic improvement of P-efficient rice cultivars.

## Materials and methods

### Plant materials and growth conditions

The experiment was conducted in a greenhouse at the Crop Physiology Laboratory of Jilin Agricultural University, Jilin, China, from April 2023 to October 2024. A total of 156 japonica rice (*Oryza sativa* L. ssp. *japonica*) germplasm resources, specifically bred and adapted for cultivation in Jilin Province, were used (**Supplementary Table 1**). These materials were provided by the Rice Research Institute of Jilin Agricultural University and had been previously evaluated in a large-scale screening program for low-P tolerance conducted by our laboratory (Zhang et al., 2025). Based on this population, four cultivars with contrasting levels of low-P tolerance were selected for mechanistic analysis: the tolerant cultivars Jinongda 873 (J873) and Jinongda 705 (J705), and the sensitive cultivars Tong 35 (T35) and Longdao 20 (L20).

To avoid interference from seed-borne microorganisms, the seeds were rigorously surface sterilized before sowing. They were first immersed in 30% H_2_O_2_ for 30 min, thoroughly rinsed with deionized water, and incubated in 0.1% NaClO for 24 h to ensure sterilization and release dormancy. The treated seeds were subsequently transferred to a constant-temperature and humidity-controlled incubator at 35 °C for germination. After radicle emergence at the white-tip stage, the seeds were sown in seedling trays filled with clean vermiculite at a density of three seeds per hill and covered with a thin vermiculite layer to maintain moisture. Following sowing, the trays were maintained in a shallow water layer for 3 d. Once the initial seedling establishment had occurred, the trays were moved to 20 L hydroponic containers. To facilitate adaptation to the hydroponic system, seedlings were first cultured in deionized water for 5 d, during which growth depended only on seed endosperm reserves. The medium was then replaced with half-strength (1/2) Kimura B nutrient solution for 7 d, followed by a full-strength (100%) solution for a further 7 d. The composition is listed in **Supplementary Table 2**. The nutrient solution was renewed every 5 d, and pH was maintained strictly between 5.5 and 6.0 by daily adjustment with 0.1 mM HCl or NaOH.

When the seedlings reached the three-leaf stage, uniform plants were selected for treatment. Two phosphorus regimes were established in the nutrient solution: (1) normal phosphorus (NP), supplied as 0.18 mM H_2_PO_4_^−^, and (2) low phosphorus (LP), supplied as 0.009 mM H_2_PO_4_^−^. This LP concentration was determined through a preliminary experiment based on relevant literature (Wang et al., 2021), as it serves as an optimal physiological threshold to effectively induce distinct P-starvation responses without causing rapid plant mortality. To preserve single-factor control, the potassium (K^+^) deficit created in the LP treatment by phosphate reduction was compensated for with an equimolar amount of KCl. All other nutrient concentrations were kept identical to those in the NP treatment.

For the main hydroponic experiment, each 20-L container was considered an independent biological replicate. Three biological replicates were established for each cultivar under each phosphorus regime, with 160 uniform plants per container. Sampling was conducted at 0, 7, 14, and 21 days after treatment (DAT). Following the principle that non-destructive measurements should precede destructive sampling, gas exchange parameters were first recorded from the uppermost fully expanded leaves of representative plants showing uniform growth. Immediately after measurement, the leaves were excised, and the midribs were removed. Each leaf sample was divided into two portions. One portion was used fresh for photosynthetic pigment extraction, while the other was rapidly frozen in liquid nitrogen and stored at −80 °C for the analysis of enzyme activities and metabolites. Simultaneously, three additional intact plants were harvested from each treatment for biomass determination.

### Determination of root phosphorus uptake kinetics

Root P uptake kinetics were assessed using a modified nutrient depletion approach. Rice seedlings grown to the three-leaf stage were first transferred to a full-strength nutrient solution (0.18 mM H_2_PO_4_^−^) and allowed to recover for 7 d to reduce variations among individuals. The recovered seedlings were assigned to two treatments. One group remained in a complete nutrient solution as the non-starved control for the determination of basal uptake activity.

The other group was subjected to phosphorus deprivation (0.00 mM H_2_PO_4_^−^) for a 48-h pre-culture period. Following conventional depletion methodologies, this duration was strictly adopted to deplete the residual internal phosphorus reserves in the seedlings. This critical step eliminates internal feedback inhibition and fully induces the root high-affinity phosphate transport system, thereby ensuring an accurate assessment of the maximum uptake potential (Zhu et al., 2019; Pereira et al., 2021). After pre-culture, the roots were thoroughly rinsed with deionized water to remove surface-adsorbed ions. The seedlings were then immediately transferred to 2-L black plastic containers containing the standard assay solution (0.18 mM H_2_PO_4_^−^) to initiate dynamic depletion measurements. The assay was conducted in a controlled growth chamber at 25 °C, 8000 lx light intensity, and 60–70% relative humidity.

Following pretreatment, seedlings were transplanted into 2-L black plastic containers filled with standard assay solution (0.18 mM H_2_PO_4_^-^). Each container was treated as one independent biological replicate, with three replicates established for each cultivar. Opaque containers were used to prevent light penetration and inhibit algal growth. Seedlings were supported on a single floating foam board per container. Each board contained five holes, with five plants placed in each hole (yielding a total of 25 plants per container), and a 2 cm spacing maintained between adjacent holes to minimize inter-root competition. During the 24 h depletion period, 5 mL nutrient solution samples were collected at 1, 2, 3, 4, 5, 6, 8, 10, 12, and 24 h. The residual P concentration in the solution was quantified dynamically using the molybdenum-antimony anti-spectrophotometric method (Murphy and Riley, 1962). To maintain an assay volume of 2 L, deionized water was replenished every 30 min throughout the measurement period. At the end of the assay, the root fresh weight of each replicate was determined precisely and used to normalize the kinetic parameters.

To elucidate the kinetic characteristics of root phosphorus (P) uptake, the time-dependent depletion of P concentration (*C*) in the nutrient solution was continuously monitored. The depletion trajectory was mathematically fitted to a quadratic polynomial equation:


$$C=a+bt+ct^{2}$$

The fitted coefficients (*a*, *b*, and *c*) were subsequently utilized to derive the instantaneous depletion rate via the first derivative (dC/dt=*b* + 2ct). Assuming that the maximum depletion rate occurs at the initial stage of the experiment (*t* = 0), the absolute value of the linear coefficient (|*b*|, representing the initial slope of the depletion curve) was used to compute the maximum P uptake rate (*I*_max_). The Michaelis constant (*K*_m_) is defined as the external P concentration at which the net uptake rate reaches half of *I*_max_ (Epstein and Hagen, 1952). was also algebraically derived based on these coefficients. The precise calculation formulas are as follows:


$$I_{max}=\frac{|b|\times V}{FRW}$$

$$K_{m}=\frac{b^{2}}{16a}-\frac{b^{2}}{4a}+c$$

where *V* represents the total volume of the nutrient solution (L), and *FRW* denotes the root fresh weight (g). In the polynomial equation, *a* is the intercept, *b* is the initial slope, and *c* is the coefficient of the quadratic term.

### Determination of dry matter accumulation and phosphorus content

At each sampling point, plants were harvested and separated into shoots and roots. After thorough rinsing with deionized water to remove surface ions, the samples were first heated at 105°C for 30 min and then dried at 80°C to a constant weight. Shoot dry weight (SDW) and root dry weight (RDW) were recorded, and the root-to-shoot ratio (R/S ratio) was calculated. The dried material was ground into fine powder and sieved, after which approximately 0.5 g of each sample was digested at a high temperature with an H_2_SO_4_–H_2_O_2_ mixture. The total phosphorus concentration was determined at 700 nm using the molybdenum-antimony anti-spectrophotometric method after cooling and dilution to a fixed volume (Lu, 1999).

### Determination of photosynthetic pigments and gas exchange parameters

Fresh leaf tissue (0.1 g) was accurately weighed, cut into small pieces, and transferred into stoppered test tubes containing 10 mL of 80% acetone. Extraction was performed in the dark at room temperature for 24 h until the leaf tissue completely lost its green color. The absorbance of the extract was measured at 663, 645, and 470 nm using a UV-visible spectrophotometer. The concentrations of chlorophyll a (*C*_a_), chlorophyll b (*C*_b_), and total chlorophyll (*C*_T_) were calculated using the equations described by Lichtenthaler (Lightenthaler, 1987).

Leaf gas exchange was measured *in situ* using a portable photosynthesis system (LI-6400XT, LI-COR, USA). Measurements were conducted between 9:00 and 11:30 AM. The chamber conditions were controlled as follows: open gas circuit, leaf chamber temperature of 25°C, photosynthetic photon flux density (PPFD) of 1000 µm m^−2^ s^−1^ generated by a red-blue LED source, and CO_2_ concentration maintained at 400 µmol mol^−1^ using a compressed gas cylinder. Once steady-state conditions were established, net photosynthetic rate (*P*_n_), stomatal conductance (*G*_s_), intercellular CO_2_ concentration (*C*_i_), and transpiration rate (*T*_r_) were recorded automatically.

### Determination of leaf carbon and nitrogen metabolites and key enzyme activities

The activities of key enzymes involved in carbon and phosphorus metabolism, including ribulose-1, 5-bisphosphate carboxylase/oxygenase (Rubisco), sucrose phosphate synthase (SPS), sucrose synthase (SS), and acid phosphatase (ACP), were quantified using specific microplate-based assay kits (Suzhou Michy Biomedical Technology Co., Ltd., Jiangsu, China; www.michyBio.com). These enzymatic activities were rigorously determined using microplate-based spectrophotometric and colorimetric methods following established protocols. Specifically, Rubisco activity was measured by continuously monitoring the oxidation rate of NADH at 340 nm using an NADH-linked coupling assay (Zelitch and Ochoa, 1953). The activities of SPS and SS were determined using the resorcinol colorimetric method at 480 nm, as described by Schrader and Saute (Schrader and Sauter, 2002). ACP activity was quantified by measuring the generation rate of p-nitrophenol at 405 nm (Liu et al., 2016). For all colorimetric assays (SPS, SS, and ACP), standard curves were constructed using the provided calibrators to ensure accurate quantification, yielding high linear regression coefficients (R^2^ ≥ 0.99). Furthermore, as a reliable internal control, all final enzymatic activities were normalized to the precise fresh weight (FW) of the leaf samples.

Carbon- and nitrogen-related metabolites were determined using standard colorimetric methods. The soluble protein content was measured using the Coomassie Brilliant Blue G-250 method (Bradford, 1976). Soluble sugar content was determined using the anthrone-sulfuric acid method (Kochert, 1978), and total free amino acids using the ninhydrin method (Bates et al., 1973). The absorbance for all enzymatic and metabolic assays was measured using a multimode microplate reader (SpectraMax i3x, Molecular Devices, LLC, Urstein, Austria).

### Statistical analysis

The experimental data were organized using Microsoft Excel 2021 and analyzed statistically using SPSS Statistics 27.0 (IBM Corp., Armonk, NY, USA). To ensure analytical precision, all biochemical assays, including enzymatic activity and metabolite quantification, were performed with three technical replicates for each independent biological sample. All results were presented as mean ± standard error (SE). For each measured trait, comparisons among the four cultivars were performed separately at each sampling time within each phosphorus treatment (NP or LP) using one-way analysis of variance (ANOVA), followed by the least significant difference (LSD) test at the 0.05 significance level. Additionally, a two-way ANOVA was performed to systematically evaluate the main effects of phosphorus regimes (P) and cultivars (C), as well as their interaction (P × C), on the physiological and phenotypic traits throughout the experimental period.

Random forest (RF) analysis was performed in R (v4.4.2) using the rfPermute and rfUtilities packages to identify the traits most strongly associated with biomass accumulation at 21 d. Model stability was improved by fixing the random seed at 500 and setting the number of trees (ntree) to 5000. Root phosphorus uptake kinetic parameters were combined with the physiological traits measured at 21 d, including leaf gas exchange parameters and carbon–nitrogen metabolic indices, to construct the predictor matrix. In comparison, biomass accumulation at 21 d was used as the response variable. Separate RF analyses were conducted for the NP and LP treatments. For both the RF and PLS-SEM analyses, each biological replicate was treated as an independent sample. Based on the variables highlighted by the RF analysis, partial least squares structural equation modeling (PLS-SEM) was conducted in R using the plspm, semPLS, plspmGUI, matrixpls, and semTools packages. A hypothetical pathway was specified to describe the relationships among root phosphorus uptake capacity, phosphorus accumulation, carbon assimilation, carbon–nitrogen metabolism, and biomass formation at 21 d. Separate models were constructed for the NP and LP treatments. The significance of path coefficients and model fit was assessed by bootstrapping (n = 5000, *P* < 0.05). These analyses were used to identify coordinated trait associations related to final biomass accumulation rather than to infer temporal dynamics during the treatment period. Data visualization was performed using Origin 2021 (OriginLab Corp., USA) and the circlize package in R.

## Results

### Genotypic differences in root phosphorus uptake kinetics

As summarized in **Table 1**, the root phosphate uptake equations demonstrated consistently high coefficients of determination (R^2^) for all cultivars and treatments. In the low-P-tolerant cultivars J873 and J705, R^2^ values ranged from 0.7767 to 0.9297, whereas in the low-P-sensitive cultivars T35 and L20, the corresponding range was 0.7424 to 0.9596. These results confirmed that the fitted equations provided a reliable description of the phosphate depletion process.

**Table 1:** Regression equations describing Pi, phosphate *C*, concentration in the depletion solution as a function of t, time for different rice varieties under contrasting phosphorus treatments.

Variety	NP	R^2^	LP	R^2^
J873	C= −0.0057t^2^+0.2137t+4.129	R^2^ = 0.7767	C= −0.004t^2^+0.1907t+2.041	R^2^ = 0.9297
	C’= −0.0114t+0.2137		C’= −0.008t+0.1907	
J705	C= −0.0069t^2^+0.2801t+4.586	R^2^ = 0.8184	C= −0.0069t^2^+0.2811t+3.055	R^2^ = 0.8952
	C’= −0.0138t+0.2801		C’= −0.00138t+0.2811	
T35	C= −0.0078t^2^+0.2912t+3.223	R^2^ = 0.9596	C= −0.0063t^2^+0.2577t+1.781	R^2^ = 0.8175
	C’= −0.0156t+0.2912		C’=−0.0126t+0.2577	
L20	C= −0.0055t^2^+0.2344t+2.839	R^2^ = 0.8398	C= −0.0059t^2^+0.2409t+2.118	R^2^ = 0.7424
	C’= −0.011t+0.2344		C’= −0.0118t+0.2409	

NP, normal phosphorus treatment; LP, low phosphorus treatment. The rice cultivars evaluated include the low-P tolerant cultivars J873, Jinongda 873 and J705, Jinongda 705, and the low-P sensitive cultivars T35, Tong 35 and L20, Longdao 20.

**Figure 1:**
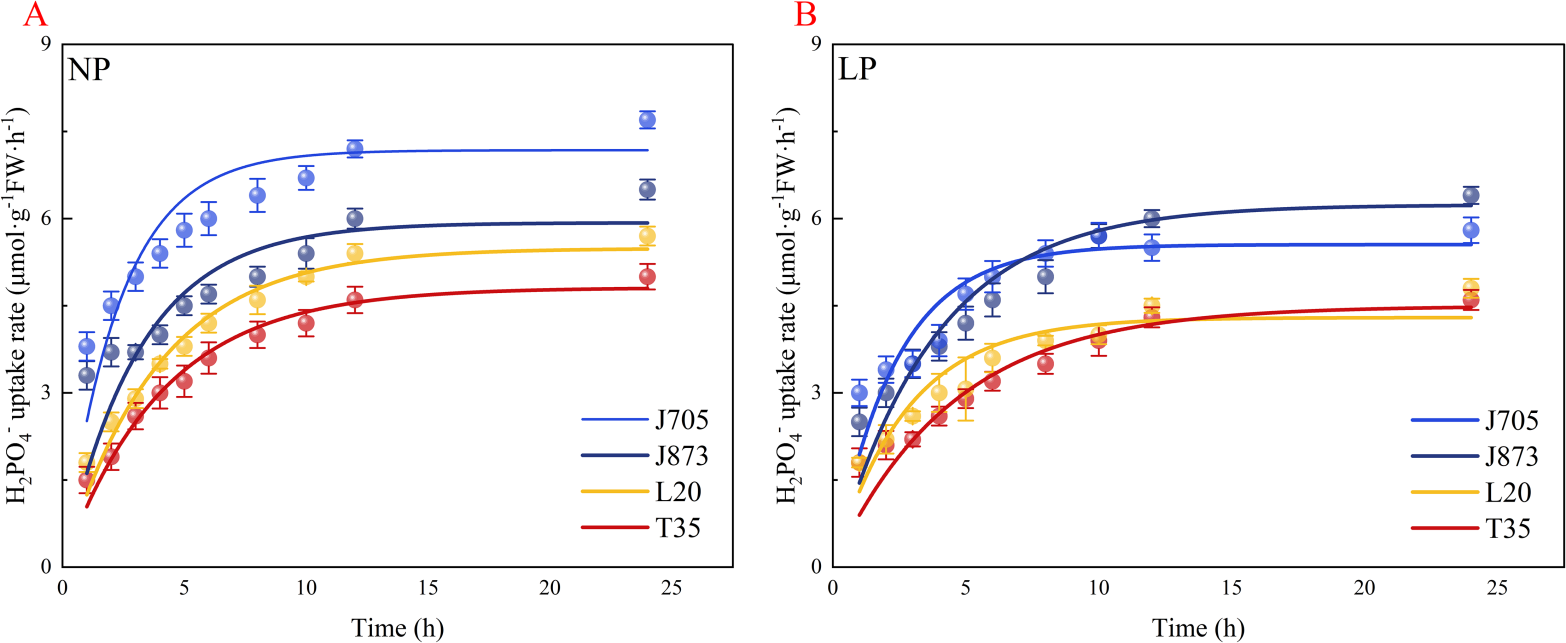
Time course of Pi, phosphate uptake rates in rice seedlings. **(A)** Uptake kinetics under constitutive conditions (NP); **(B)** uptake kinetics under inducible conditions (LP). Seedlings were supplied with normal phosphorus (NP, 0.18 mM H_2_PO_4_^−^) or subjected to phosphorus starvation for 48 h (LP, 0 mM H_2_PO_4_^−^). Data are presented as mean ± SE (n = 3). The rice cultivars evaluated include the low-P-tolerant cultivars J873, Jinongda 873 and J705, Jinongda 705, and the low-P-sensitive cultivars T35, Tong 35 and L20, Longdao 20.

**Figure 1** illustrates that the P uptake curves of all four cultivars closely followed Michaelis–Menten kinetics and displayed a typical saturation pattern within 24 h. Under NP conditions, a significant genotypic divergence in uptake dynamics was observed. J873 exhibited a relatively gradual increase in the P uptake rate before approaching a plateau, whereas J705 responded more abruptly, with a rapid rise during the first 3 h, followed by progressive deceleration and stabilization after 12 h. In contrast, both sensitive cultivars, T35 and L20, maintained slower increases in the uptake rate and did not level off until after 12 h. At 12 h, the uptake rate of T35 was significantly lower than those of J873 and J705 by 20.6% and 34.2%, respectively, whereas L20 showed corresponding reductions of 9.5% and 25.0%.

A faster kinetic response was induced in the tolerant cultivars under LP treatment. In both J873 and J705, the P uptake rates rose sharply during the initial 4–5 h and then continued to increase more gradually before reaching saturation after 12 h. In contrast, T35 and L20 still displayed only a slow upward trend and similarly plateaued after 12 h. At 12 h, the uptake rate of T35 remained significantly below those of J873 and J705 by 29.7% and 22.4%, respectively, while the corresponding decreases in L20 were 25.0% and 17.2%.

As presented in **Table 2**, J705 presented the greatest maximum uptake rate (*I*_max_), reaching 6.936 µmol·g^−1^ root FW·h^−1^, whereas the lowest value was recorded for T35 at 4.159 µmol·g^−1^ root FW·h^−1^. When averaged across genotypes, the low-P-tolerant cultivars exceeded the sensitive cultivars in *I*_max_ by 15.2% under NP and by 26.23% under LP. In contrast, *K*_m_ exhibited the opposite pattern. Under NP conditions, the ranking of H_2_PO_4_^−^ uptake capacity based on *I*_max_ was J705 > J873 > T35 > L20, indicating that the tolerant cultivars combined a higher uptake potential with a stronger affinity than the sensitive cultivars. Under LP treatment, the order shifted slightly to J705 > J873 > L20 > T35.

**Table 2:** Kinetic parameters of phosphate (H_2_PO_4_^−^) uptake in rice seedlings under contrasting phosphorus treatments.

Variety	Treatment	*I*_max_ (µmol·g^−1^ Root FW·h^−1^)	*K*_m_ (µmol·L^−1^)
J873	NP	5.825	0.4942
	LP	5.166	1.077
J705	NP	6.936	0.3751
	LP	5.604	0.6267
T35	NP	5.791	0.521
	LP	4.159	1.552
L20	NP	5.284	1.061
	LP	4.373	1.358

Kinetic parameters were derived from the average phosphate depletion curve across three independent biological replicates (n = 3). NP, normal phosphorus treatment; LP, low phosphorus treatment. The rice cultivars include the low-P-tolerant cultivars J873, Jinongda 873 and J705, Jinongda 705, and the low-P-sensitive cultivars T35, Tong 35 and L20, Longdao 20. *I*_max_ denotes the maximum phosphate uptake rate of the root transport system. *K*_m_ denotes the Michaelis constant, defined as the external phosphate concentration at which the net uptake rate reaches half of *I*_max_.

### Effects of low phosphorus treatment on biomass and P accumulation

**Figure 2:**
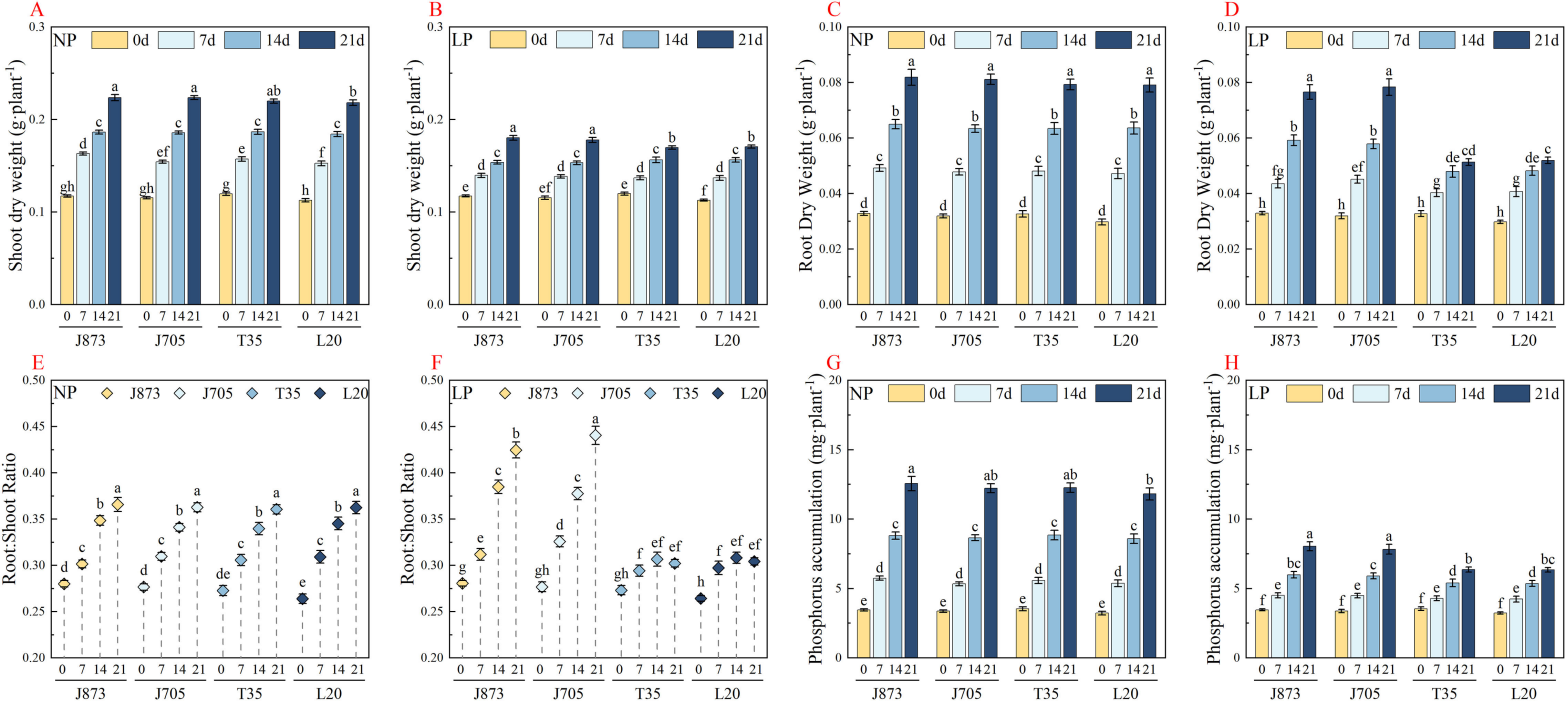
Temporal changes in biomass accumulation and P, phosphorus traits of rice seedlings under different P supplies. **(A, B)** Shoot dry weight; **(C, D)** root dry weight; **(E, F)** root-to-shoot ratio; **(G, H)** total P accumulation. Treatments included normal phosphorus (NP, 0.18 mM H_2_PO_4_^−^) and low phosphorus (LP, 0.009 mM H_2_PO_4_^−^). Data are presented as mean ± SE (n=3). Different lowercase letters indicate significant differences across all treatments, time points, and cultivars (*P* < 0.05). The rice cultivars evaluated include the low-P-tolerant cultivars J873, Jinongda 873 and J705, Jinongda 705, and the low-P-sensitive cultivars T35, Tong 35 and L20, Longdao 20. The numbers 0, 7, 14, and 21 refer to the incubation time (days) under the respective phosphorus treatments.

Phosphorus supply exerted a strong influence on biomass formation in rice seedlings. Under NP conditions, all four cultivars followed similar growth trajectories (**Figure 2A**). In contrast, LP treatment substantially suppressed growth and revealed genotypic divergence in growth performance (**Figure 2B**). By day 21, the low-P-tolerant cultivars J873 and J705 had established a distinct growth advantage. Their shoot dry weight (SDW) was significantly higher than that of the sensitive cultivars T35 and L20, with differences of 5.45% and 4.85%, respectively (*P* < 0.05). No significant difference was detected between the two tolerant cultivars, indicating a shared capacity to preserve shoot biomass under phosphorus deficiency. Furthermore, after 21 days of phosphorus stress treatment, two-way ANOVA confirmed highly significant main effects of phosphorus and cultivar on shoot dry weight, with no significant interaction (**Supplementary Table 3**).

Root growth responded differently to shoot biomass. Under NP treatment, the root dry weight (RDW) progressively increased in all cultivars (**Figure 2C**). Although LP exposure also constrained root growth, the inhibitory effect was weaker in roots than in that in shoots (**Figure 2D**). More importantly, J873 and J705 maintained stronger root development under LP, with significantly greater RDW than the sensitive cultivars on days 14 and 21 (*P* < 0.05). This pattern indicates that tolerant genotypes preferentially sustain root biomass production under low-P conditions, thereby reinforcing nutrient foraging capacity. For root dry weight, after 21 days of phosphorus stress treatment, two-way ANOVA revealed highly significant main and interaction effects of phosphorus and cultivar, indicating distinct root growth responses among the tested cultivars under low-phosphorus conditions (**Supplementary Table 3**).

Biomass partitioning was altered by phosphorus availability. Under NP conditions, the root-to-shoot ratio (R/S) remained comparatively stable among cultivars (**Figure 2E**). Under LP treatment, R/S increased markedly and reached its highest values in the tolerant cultivars J873 and J705 on day 21 (**Figure 2F**). At this stage, the R/S ratios of the tolerant genotypes exceeded those of T35 and L20 by approximately 43.11% and 42.18%, respectively (*P* < 0.05). These results indicate that tolerant cultivars possess a stronger capacity to reallocate biomass to roots under phosphorus deficiency, thereby supporting adaptive root growth. Regarding the root-to-shoot ratio, after 21 days of phosphorus stress treatment, two-way ANOVA confirmed a highly significant cultivar effect and a strong interaction, despite a non-significant main effect of phosphorus, highlighting distinct biomass allocation strategies among the cultivars (**Supplementary Table 3**).

As a direct indicator of phosphorus acquisition capacity, total phosphorus accumulation (PA) also exhibited a clear genotypic response. Under NP conditions, PA increased steadily in all cultivars (**Figure 2G**). Although LP treatment reduced overall phosphorus accumulation, tolerant genotypes consistently maintained a clear advantage (**Figure 2H**). On day 21, PA in J873 and J705 was approximately 24.61% and 25.00% higher, respectively, than that in the sensitive cultivars T35 and L20 (*P* < 0.05). This result confirmed that the tolerant genotypes retained higher phosphorus acquisition efficiency and maintained a more favorable P nutritional status under P-deficient conditions. For phosphorus accumulation, after 21 days of phosphorus stress treatment, two-way ANOVA revealed highly significant main effects and a significant interaction, indicating varied phosphorus uptake capacities among the tested cultivars under stress (**Supplementary Table 3**).

### Responses of photosynthetic pigments and gas exchange parameters

**Figure 3:**
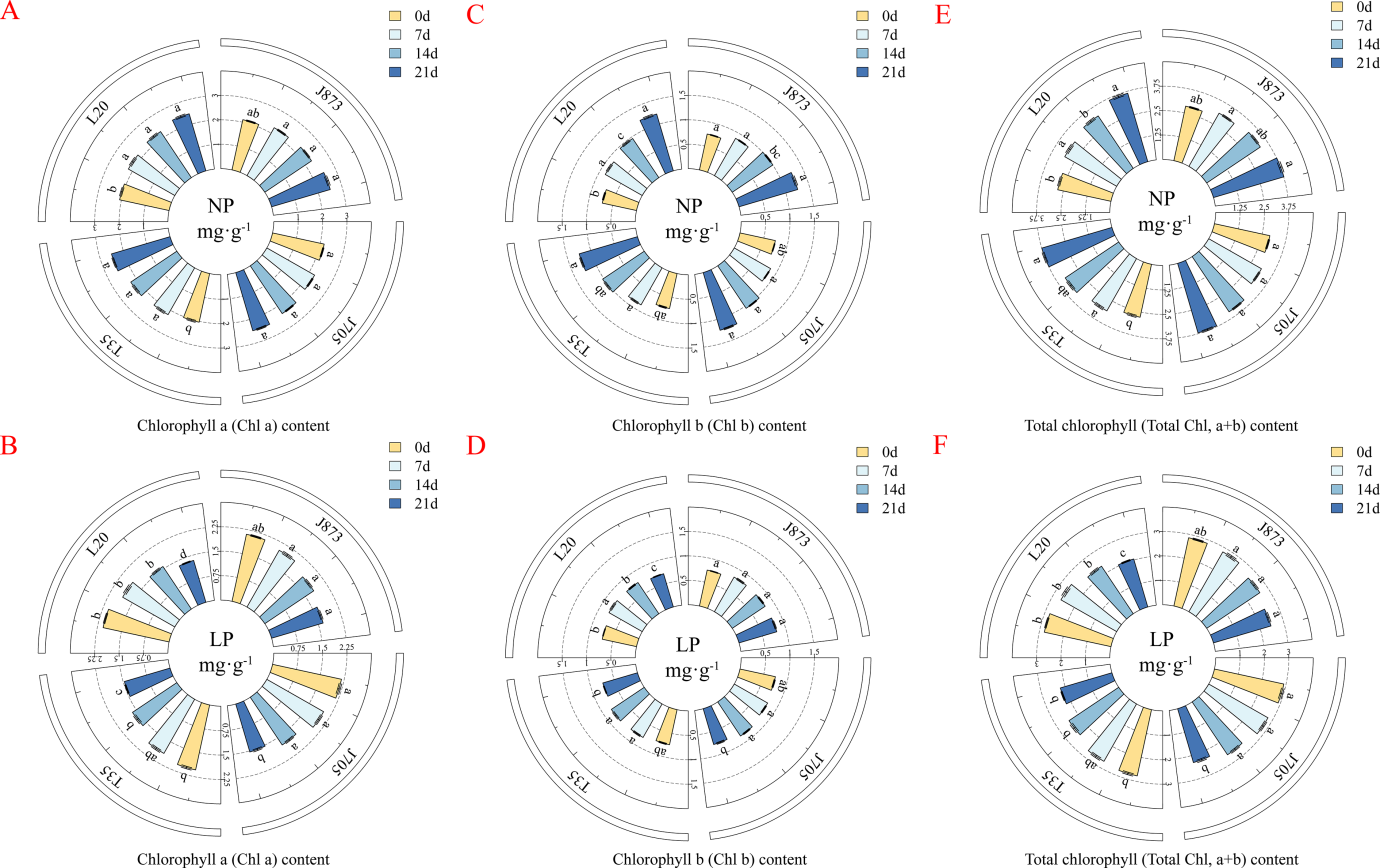
Chlorophyll contents of rice seedlings under different P supplies. **(A, B)** Chl a, Chlorophyll a content; **(C, D)** Chl b, chlorophyll b content; **(E, F)** total chlorophyll (Total Chl, a+b) content. Treatments: NP (0.18 mM H_2_PO_4_^−^) and LP (0.009 mM H_2_PO_4_^−^). Data are presented as mean ± SE (n=3). Different lowercase letters indicate significant differences among varieties at each sampling point (*P* < 0.05). The rice cultivars evaluated include the low-P-tolerant cultivars J873, Jinongda 873 and J705, Jinongda 705, and the low-P-sensitive cultivars T35, Tong 35 and L20, Longdao 20. The numbers 0, 7, 14, and 21 refer to the incubation time (days) under the respective phosphorus treatments.

Phosphorus availability significantly influenced photosynthetic pigment content. Under NP conditions, chlorophyll a (Chl a) levels increased uniformly across all varieties (**Figure 3A**). In contrast, the LP treatment induced a significant decline in Chl a content over time (**Figure 3B**). However, tolerant cultivars (J873 and J705) exhibited superior retention capacities. By day 21, their Chl a content was significantly higher, ranging from approximately 8.83% to 18.97%, compared to sensitive varieties (T35 and L20; *P* < 0.05). This indicates that tolerant genotypes can effectively mitigate the pigment degradation induced by the LP treatment. Under NP conditions, Chlorophyll b (Chl b) content increased consistently with no significant genotypic differences (**Figure 3C**). The LP treatment induced a significant reduction (**Figure 3D**), yet tolerant varieties demonstrated a clear advantage. By day 21, J873 and J705 maintained significantly higher Chl b levels, ranging from approximately 5.89% to 10.83%, compared to sensitive cultivars (*P* < 0.05). Notably, J873 exhibited significantly higher Chl b retention than J705 (*P* < 0.05). This suggests that while both are tolerant, J873 possesses a superior capacity to maintain the stability of light-harvesting complexes under P-deficient conditions. For photosynthetic pigments, after 21 days of phosphorus stress treatment, two-way ANOVA revealed highly significant main effects of both phosphorus and cultivar on Chl a and Chl b. Notably, a highly significant interaction was observed for Chl a, indicating distinct response patterns among cultivars, whereas the interaction for Chl b was not significant, suggesting a consistent response across all tested cultivars (**Supplementary Table 3**).

Total chlorophyll content (Total Chl) serves as a key indicator of photosynthetic potential. Under NP conditions, Total Chl increased consistently across all varieties, showing no significant differences at day 21 (**Figure 3E**). The LP treatment significantly restricted accumulation (**Figure 3F**); however, tolerant varieties were notably less affected. By day 21, J873 and J705 maintained significantly higher Total Chl levels approximately 7.85%–16.15% higher compared to sensitive varieties (*P* < 0.05). The maintenance of higher total chlorophyll levels in tolerant varieties under the LP treatment provides the material basis for sustaining their superior photosynthetic capacity. For Total Chl, after 21 days of phosphorus stress treatment, two-way ANOVA confirmed highly significant main and interaction effects of both phosphorus and cultivar (**Supplementary Table 3**).

**Figure 4:**
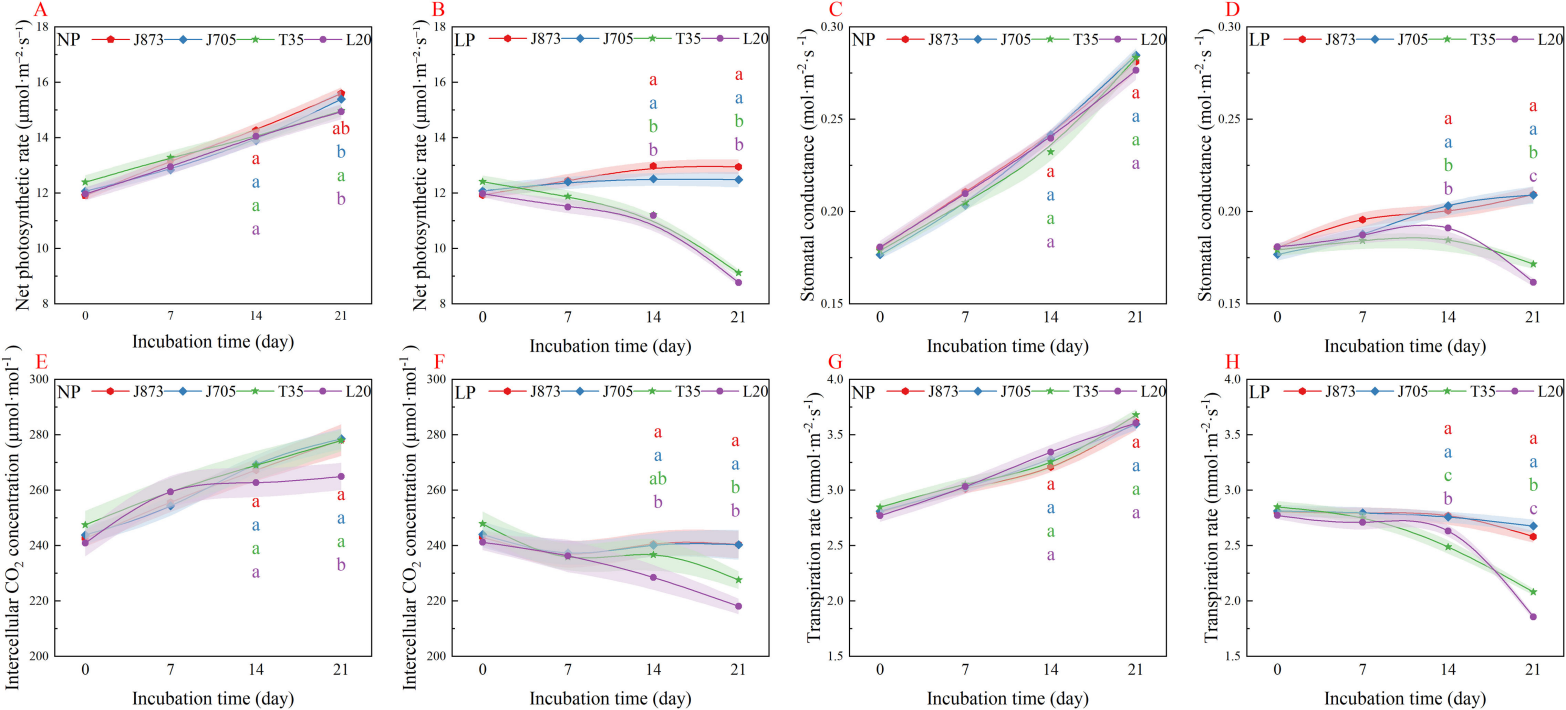
Temporal changes in photosynthetic gas exchange parameters of rice seedlings under different phosphorus treatments. **(A, B)** Net *P*_n_, photosynthetic rate; **(C, D)** *G*_s_, stomatal conductance; **(E, F)** *C*_i_, intercellular CO_2_ concentration (); **(G, H)** *T*_r_, transpiration rate. Treatments: NP (0.18 mM H_2_PO_4_^−^) and LP (0.009 mM H_2_PO_4_^−^). Solid lines represent mean values, and shaded ribbons indicate the standard error (SE; n = 3). Note: The rice cultivars evaluated include the low-P-tolerant cultivars J873, Jinongda 873 and J705, Jinongda 705, and the low-P-sensitive cultivars T35, Tong 35 and L20, Longdao 20. The numbers 0, 7, 14, and 21 denote the incubation time (days) under the respective phosphorus treatments. Different-coloured lowercase letters at specific time points indicate significant differences among the corresponding cultivars (*P* < 0.05).

The net photosynthetic rate (*P*_n_) serves as a direct indicator of carbon assimilation capacity. Under NP conditions, *P*_n_ exhibited a consistent increasing trend across all varieties (**Figure 4A**). In contrast, the LP treatment induced divergent responses (**Figure 4B**). As the culture progressed, a clear separation of the curves was observed, with the non-overlapping shaded bands (representing standard error) visually highlighting the distinct advantage of tolerant varieties. By day 21, J873 and J705 maintained significantly higher *P*_n_ values, approximately 39.47%–45.09% higher compared to sensitive cultivars (*P* < 0.05). This divergence suggests that tolerant genotypes can effectively sustain higher photosynthetic rates during the later stages of the LP treatment.

Stomatal conductance (*G*_s_) acts as a critical regulator of gas exchange. Under NP conditions, *G*_s_ exhibited a consistent increasing trend across all varieties (**Figure 4C**). The LP treatment resulted in a significant reduction; however, genotypic divergence became increasingly evident over time (**Figure 4D**). By days 14 and 21, the curves of tolerant varieties clearly separated from those of sensitive ones. Consequently, J873 and J705 maintained significantly higher *G*_s_ values by day 21, approximately 21.95%–29.33% higher compared to sensitive counterparts (T35 and L20; *P* < 0.05). This suggests that tolerant varieties can effectively maintain stomatal aperture under the LP treatment, thereby facilitating the entry of photosynthetic substrates.

Intercellular CO_2_ concentration (*C*_i_) directly determines substrate availability for carbon assimilation. Under the NP treatment, *C*_i_ increased steadily across all varieties, although L20 exhibited consistently lower levels (**Figure 4E**). The LP treatment induced a significant reduction in *C*_i_, signaling a pronounced stomatal limitation (**Figure 4F**). However, tolerant varieties were notably less affected, maintaining trajectories consistently above those of sensitive cultivars. By day 21, J873 and J705 maintained significantly higher *C*_i_ values, approximately 5.63%–10.22% higher compared to sensitive varieties (T35 and L20; *P* < 0.05). These findings suggest that tolerant genotypes effectively alleviate intercellular carbon starvation under the LP treatment by maintaining superior stomatal status.

The transpiration rate (*T*_r_) reflects plant water status and drives nutrient transport. Under NP conditions, *T*_r_ increased comparably across all varieties (**Figure 4G**). The LP treatment led to a gradual decline; however, genotypic differences became increasingly pronounced (**Figure 4H**). After day 14, the non-overlapping shaded bands (representing standard error) visually highlighted the distinct advantage of tolerant varieties. By day 21, J873 and J705 maintained significantly higher *T*_r_ values, approximately 26.31%–41.64% higher compared to sensitive varieties (T35 and L20; *P* < 0.05). This higher transpiration rate facilitates water balance maintenance and drives root-to-shoot nutrient transport under the LP treatment.

For leaf gas exchange parameters, after 21 days of phosphorus stress, two-way ANOVA revealed highly significant main effects of phosphorus and cultivar across all measured traits (*P*_n_, *G*_s_, *C*_i_, and *T*_r_). Notably, highly significant interactions were observed for *P*_n_, *G*_s_, and *T*_r_, indicating strong cultivar-specific photosynthetic adjustments, whereas the interaction for *C*_i_ was not statistically significant (**Supplementary Table 3**).

### Responses of key enzymes and metabolites in carbon and nitrogen metabolism

**Figure 5:**
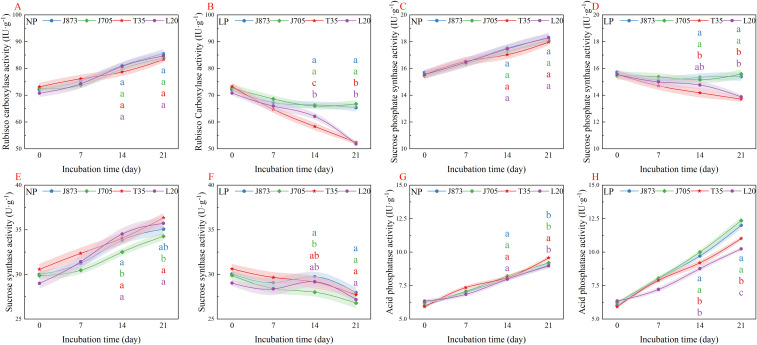
Temporal changes in enzymatic activities related to carbon and phosphorus metabolism in rice seedlings under different phosphorus treatments. **(A, B)** Ribulose-1, 5-bisphosphate carboxylase (Rubisco) activity; **(C, D)** SPS, sucrose phosphate synthase activity; **(E, F)** SS, sucrose synthase activity; **(G, H)** ACP, acid phosphatase activity. Treatments: NP (0.18 mM H_2_PO_4_^−^) and LP (0.009 mM H_2_PO_4_^−^). Solid lines represent mean values, and shaded ribbons indicate the standard error (SE; n = 3). The rice cultivars evaluated include the low-P-tolerant cultivars J873, Jinongda 873 and J705, Jinongda 705, and the low-P-sensitive cultivars T35, Tong 35 and L20, Longdao 20. The numbers 0, 7, 14, and 21 denote the incubation time (days) under the respective phosphorus treatments. Different-coloured lowercase letters at specific time points indicate significant differences among the corresponding cultivars (*P* < 0.05).

Phosphorus availability significantly influenced key enzymatic activities. Rubisco, the pivotal driver of carbon assimilation, exhibited a consistent increasing trend across all varieties under NP conditions (**Figure 5A**). The LP treatment inhibited activity, leading to a gradual decline; however, genotypic divergence became pronounced in the later stages (**Figure 5B**). By days 14 and 21, a distinct separation between the curves of tolerant and sensitive varieties was observed. Consequently, J873 and J705 maintained significantly higher Rubisco activity by day 21, approximately 26.18%–27.51% higher compared to sensitive counterparts (*P* < 0.05). This indicates that tolerant varieties can effectively maintain carboxylation efficiency under the LP treatment, ensuring the progression of photosynthesis.

Sucrose phosphate synthase (SPS) and sucrose synthase (SS) coordinately regulate photosynthate metabolism. Under NP conditions, the activities of both enzymes increased concurrently with culture duration, showing no significant genotypic differences (**Figures 5C, E**). While the LP treatment inhibited both enzymes, distinct genotypic responses were observed for SPS (**Figure 5D**). Tolerant varieties exhibited superior maintenance capacity, with shaded bands ceasing to overlap after day 14. By day 21, J873 and J705 maintained significantly higher SPS activity, approximately 12.97%–11.59% higher compared to sensitive counterparts (T35 and L20; *P* < 0.05). Conversely, SS activity exhibited no significant genotypic differences (*P* > 0.05; **Figure 5F**). These results suggest that tolerant varieties primarily promote sucrose synthesis and ensure transport to sink organs by maintaining higher SPS activity under the LP treatment.

Acid phosphatase (ACP) plays a critical role in intracellular P remobilization. Under NP conditions, ACP activity increased steadily across all varieties (**Figure 5G**). Distinct from carbon metabolism enzymes, the LP treatment further induced ACP activity, with a significantly stronger response in tolerant genotypes (**Figure 5H**). By day 21, J873 and J705 exhibited markedly higher activity, ranging from approximately 10.5% to 18.85% higher compared to sensitive varieties (T35 and L20; *P* < 0.05). This elevated ACP activity indicates that tolerant varieties possess a robust capacity for P recycling and remobilization, facilitating adaptation to external P scarcity under the LP treatment.

For the key enzymes involved in carbon and phosphorus metabolism (Rubisco, SPS, SS, and ACP), two-way ANOVA revealed highly significant main effects of phosphorus after 21 days of stress treatment. Notably, highly significant cultivar and interaction effects were observed for Rubisco, SPS, and ACP, indicating distinct enzymatic regulatory strategies among the cultivars. In contrast, although SS showed significant main effects for both factors, its interaction was not statistically significant (**Supplementary Table 3**).

**Figure 6:**
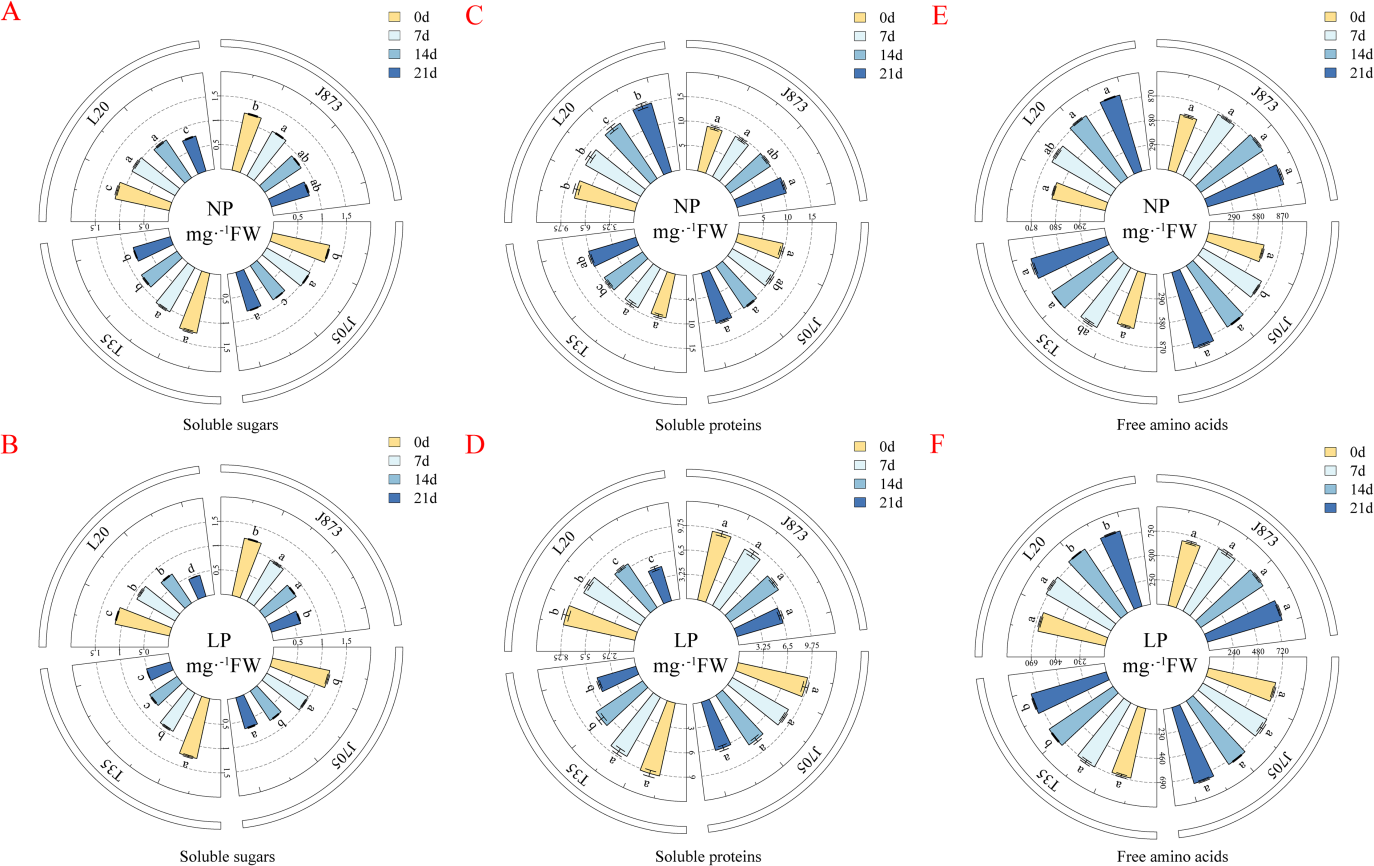
Contents of soluble sugars, soluble proteins, and free amino acids in rice seedlings under different phosphorus treatments. **(A, B)** Soluble sugars; **(C, D)** soluble proteins; **(E, F)** free amino acids. Treatments: NP (0.18 mM H_2_PO_4_^−^) and LP (0.009 mM H_2_PO_4_^−^). Values are expressed as mg·g^−1^ FW. Data are presented as mean ± SE (n=3). Different lowercase letters indicate significant differences among varieties at each sampling point (*P* < 0.05). The rice cultivars evaluated include the low-P-tolerant cultivars J873, Jinongda 873 and J705, Jinongda 705, and the low-P-sensitive cultivars T35, Tong 35 and L20, Longdao 20. The numbers 0, 7, 14, and 21 refer to the incubation time (days) under the respective phosphorus treatments.

Phosphorus availability significantly influenced soluble sugar content (SSC), a key regulator of cellular osmotic potential. Under NP conditions, SSC exhibited a gradual declining trend across all varieties, with tolerant genotypes maintaining slightly higher levels (approximately 4.06%–10.32%) by day 21 (**Figure 6A**). The LP treatment exacerbated this decline; however, tolerant varieties exhibited a superior capability to sustain SSC levels (**Figure 6B**). By day 21, J873 and J705 maintained significantly higher SSC ranging from approximately 26.87% to 42.69% compared to sensitive varieties (T35 and L20; *P* < 0.05). This suggests that tolerant genotypes effectively lower cellular osmotic potential to enhance water retention capacity under the LP treatment.

Soluble protein (SP) functions as a critical metabolic substrate and enzymatic pool. Under NP conditions, SP content increased steadily, with tolerant varieties significantly exceeding L20 (approximately 10.45%–12.54%; **Figure 6C**). The LP treatment reversed this trend, leading to a gradual decline over time (**Figure 6D**). However, tolerant varieties exhibited a superior capability to sustain SP levels. By day 21, J873 and J705 maintained significantly higher SP content, ranging from approximately 31.80% to 62.55%, compared to sensitive varieties (*P* < 0.05). This indicates that tolerant genotypes can effectively maintain protein levels under the LP treatment, ensuring the continuity of essential metabolic activities.

Free amino acids (FAA) function in osmotic adjustment and nitrogen cycling. Under NP conditions, FAA content increased gradually across all varieties, with no significant genotypic differences (**Figure 6E**). The LP treatment induced further accumulation in all genotypes; however, tolerant varieties exhibited a significantly stronger response (**Figure 6F**). By day 21, J873 and J705 maintained the highest FAA levels approximately 6.63%–6.55% higher compared to sensitive varieties (*P* < 0.05). This elevated concentration aids in maintaining cellular turgor pressure and serves as a nitrogen reservoir for remobilization under the LP treatment.

For key soluble metabolites (soluble sugars, soluble proteins, and free amino acids), two-way ANOVA after 21 days of phosphorus stress treatment revealed highly significant main effects of both phosphorus and cultivar across all three traits. Furthermore, highly significant interactive effects were observed for soluble sugars and soluble proteins, and a significant interaction was observed for free amino acids, highlighting distinct metabolic adaptation and osmotic adjustment strategies among the tested cultivars (**Supplementary Table 3**).

### Identification of key physiological factors and path analysis

**Figure 7:**
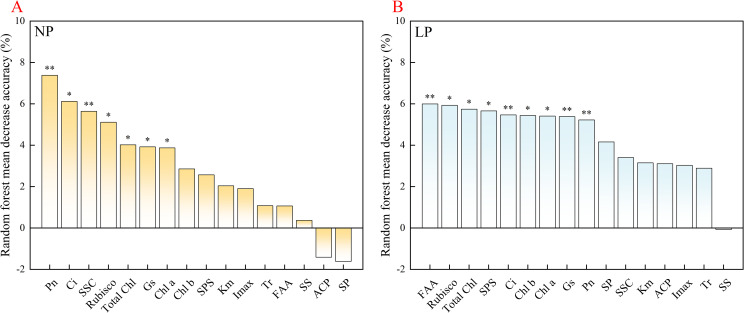
Variable importance of physiological traits in predicting biomass accumulation in rice seedlings under contrasting phosphorus treatments. **(A)** NP treatment; **(B)** LP treatment. Variable importance was assessed by MDA, mean decrease in accuracy. Treatments: NP (0.18 mM H_2_PO_4_^−^) and LP (0.009 mM H_2_PO_4_^−^). *P*_n_, net photosynthetic rate; *C*_i_, intercellular CO_2_ concentration; SSC, soluble sugar content; Rubisco, ribulose-1, 5-bisphosphate carboxylase activity; Total Chl, total chlorophyll content; *G*_s_, stomatal conductance; Chl a/b, chlorophyll a/b ratio; SPS, sucrose phosphate synthase activity; *K*_m_, Michaelis constant; *I*_max_, maximum uptake rate; *T*_r_, transpiration rate; FAA, free amino acid content; SS, sucrose synthase activity; ACP, acid phosphatase activity; SP, soluble protein content. Note: These abbreviations are used consistently across all subsequent figures and throughout the text. * *P* < 0.05 and ** *P* < 0.01 indicate significant importance based on permutation tests.

RF analysis was applied to rank the contribution of physiological traits and identify the principal predictors of low-P tolerance (**Figure 7**). Under NP conditions (**Figure 7A**), *P*_n_ produced the largest Mean Decrease in Accuracy, indicating that it was the dominant variable discriminating growth potential among genotypes. The next most influential variables were *C*_i_, SSC, and Rubisco activity. These results indicate that when the phosphorus supply was sufficient, the inter-varietal physiological divergence was governed primarily by carbon assimilation capacity.

A pronounced reorganization of predictor importance was observed under LP treatment (**Figure 7B**). FAA emerged as the top-ranking variable with the highest predictive importance, followed by Rubisco, Total Chl, and SPS. Although *P*_n_ retained its predictive value, its relative contribution declined markedly compared with that under NP conditions. Overall, the RF model revealed that the primary predictors shifted from gas exchange traits centered on *P*_n_ under NP to metabolic and enzymatic variables represented by FAA, Rubisco, and SPS under LP. This statistical pattern suggests that under phosphorus deficiency, traits associated with nitrogen metabolic adjustment, preservation of photosynthetic enzymatic function, and maintenance of sucrose synthesis capacity became stronger predictors of biomass formation than the photosynthetic rate itself, highlighting their close association with genotypic variation in low-P tolerance.

**Figure 8:**
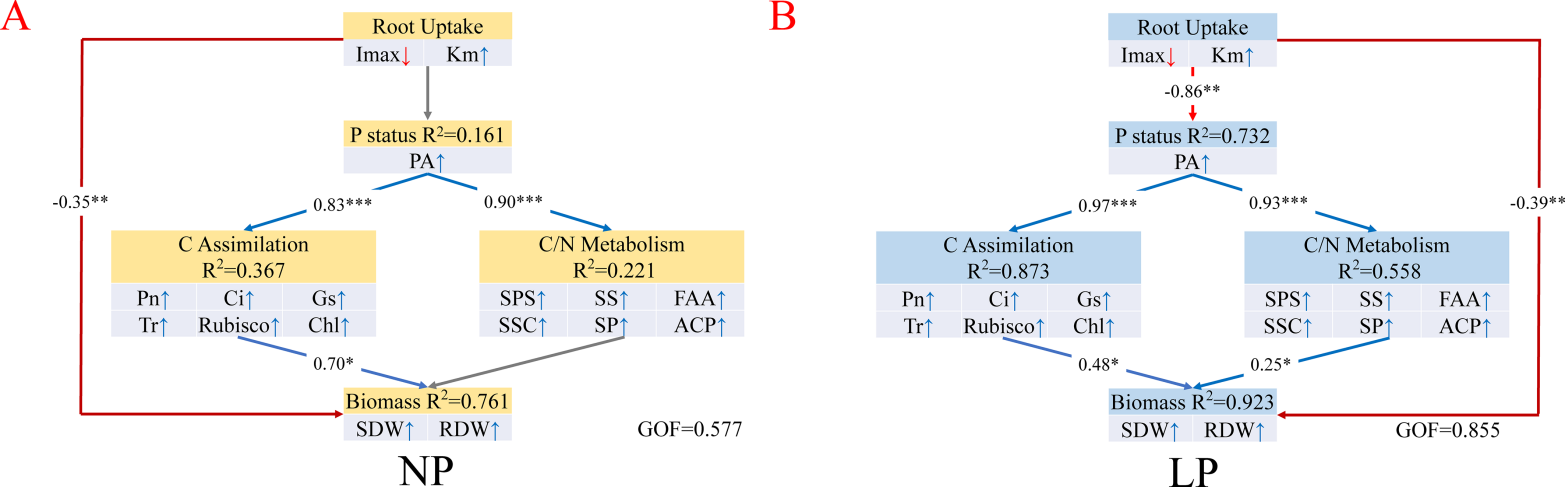
Partial least squares path modeling (PLS-PM) of physiological pathways driving biomass accumulation in rice seedlings under contrasting phosphorus treatments. **(A)** NP treatment (GoF = 0.577); **(B)** LP treatment (GoF = 0.855). Numbers on arrows indicate standardized path coefficients. The GoF index represents the overall predictive power of the model. Treatments: NP (0.18 mM H_2_PO_4_^−^) and LP (0.009 mM H_2_PO_4_^−^). GoF, Goodness of Fit; SDW, shoot dry weight; RDW, root dry weight. For other physiological parameters, refer to the abbreviations list in **Figure 7**. * *P* < 0.05, ** *P* < 0.01, and *** *P* < 0.001 indicate significant relationships. Solid blue and red lines represent significant positive and negative paths, respectively, whereas gray dashed lines represent non-significant paths (*P* > 0.05).

To explore the structural interrelationships among root uptake, phosphorus status, carbon assimilation, and carbon–nitrogen (C/N) metabolism regarding biomass formation, a Partial Least Squares Path Model (PLS-PM) was established (**Figure 8**). The model performance differed markedly between phosphorus regimes. The LP model yielded a Goodness of Fit (GOF) of 0.855, exceeding that of the NP model (0.577), and showed greater explained variance (R^2^) for biomass, carbon assimilation, and C/N metabolism. These statistical parameters suggest that the core physiological components became more strongly interdependent under LP.

Under NP conditions (**Figure 8A**), variance in biomass accumulation was primarily explained by photosynthetic carbon assimilation, which exhibited a strong direct path coefficient (0.70*). In contrast, the direct path from C/N metabolism was not significant. This statistical pathway was substantially reconfigured following LP treatment (**Figure 8B**). Root uptake traits showed a strong negative path toward phosphorus status (−0.86**), highlighting the limiting role of uptake kinetics. Concurrently, the primary predictors of biomass shifted from a single assimilation-dominated path to a dual pathway involving both carbon assimilation (0.48*) and C/N metabolism (0.25*).

Overall, the PLS-PM analysis demonstrated that under LP conditions, biomass maintenance in rice seedlings was statistically linked to the joint contributions of root phosphorus acquisition, internal phosphorus status, and shoot metabolic adjustment. The model highlights that, alongside photosynthetic carbon supply, the coordinated remodeling of C/N metabolism is an indispensable physiological component for predicting growth under phosphorus deficiency.

## Discussion

### Genotypic differences in root phosphorus uptake kinetics and acquisition capacity

P is a key mineral nutrient limiting plant growth, and the capacity of roots to acquire P from the external medium directly determines plant P nutritional status (Vance et al., 2003; De Bauw et al., 2019). Using the Claassen-Barber nutrient depletion model, this study demonstrated that LP treatment substantially reshaped the uptake kinetics of rice roots (Claassen and Barber, 1974). The tolerant cultivars J873 and J705 displayed a kinetic profile under LP, characterized by higher *I*_max_ and lower *K*_m_. From a physiological perspective, *I*_max_ reflects the upper limit of root uptake capacity under substrate-sufficient conditions (York et al., 2016). This parameter can be significantly determined by the abundance of functional phosphate transporters in the plasma membrane of root epidermal cells and the intensity of starvation-induced activation under LP. Therefore, a higher *I*_max_ provides tolerant genotypes with a stronger compensatory uptake potential when roots encounter localized P-rich zones or fluctuations in external P availability (Jackson et al., 1990; Hodge, 2004). In contrast, *K*_m_ serves as an inverse measure of transporter affinity for phosphate. A lower *K*_m_ indicates the activation of a more effective high-affinity transport system, allowing tolerant genotypes to maintain net influx even when solution P remains extremely low. This combination of high uptake capacity and high affinity represents a clear kinetic advantage, and its significance is further reflected at the whole-plant level (Yam-Chimal et al., 2022). The markedly higher shoot P accumulation maintained by the tolerant cultivars under LP treatment provides direct evidence for the effective operation of this uptake strategy (**Figure 2H**). The superior membrane transport capacity can be translated into a measurable whole-plant advantage in P interception and acquisition. This result is also consistent with earlier molecular evidence showing that tolerant crops can reconfigure uptake kinetics through the induced expression of high-affinity phosphate transporters, such as members of the *OsPT* family (Jia et al., 2011; Ye et al., 2017; Kuppe et al., 2022). Further support is provided by the PLS-PM results (**Figure 8B**), in which root uptake kinetic traits exerted a strong negative path coefficient on internal P status. Collectively, these findings indicate that under P-deficient conditions, the improvement of root uptake efficiency is not only the physiological foundation of P homeostasis maintenance but also the primary limiting process governing plant adaptation to P shortage.

### Protective roles of stomatal regulation and enzyme activity maintenance on photosynthetic capacity under low phosphorus treatment

Photosynthesis is the primary material basis for crop biomass production and is highly vulnerable to phosphorus deficiency (Carstensen et al., 2018). In the present study, although LP treatment reduced *P*_n_ in all cultivars, tolerant genotypes retained a markedly greater capacity to preserve photosynthetic function. At the stomatal level, tolerant cultivars maintained significantly higher *G*_s_ and *T*_r_. This response is essential for preserving the transpiration stream. As xylem-mediated long-distance P transport largely depends on transpiration-driven mass flow, a higher transpiration rate facilitates the upward translocation of the limited P absorbed by roots. This process provides an important physical basis for maintaining shoot P nutrition (Meng and Fricke, 2017; Li et al., 2021). To distinguish whether stomatal or non-stomatal limitations drove the LP-induced decline in *P*_n_, we evaluated the dynamic changes in intercellular CO_2_ concentration (*C*_i_). According to the classic criteria (Farquhar and Sharkey, 1982), if stomatal closure is the primary constraint, CO_2_ depletion would lead to a marked decrease in *C*_i_. However, in this study, the substantial reductions in *P*_n_ and *G*_s_ under LP were not accompanied by a strong decline in *C*_i_. This decoupling indicates that non-stomatal (biochemical) limitations predominated in restricting photosynthesis—our biochemical results further support this interpretation. LP accelerates chlorophyll degradation and destabilizes light-harvesting complexes (Hussain et al., 2021; Gao et al., 2022). In the present study, tolerant genotypes exhibited a clear delay in leaf senescence (**Figure 3F**). The maintenance of a higher total chlorophyll content supports more efficient light capture. It sustains the generation of ATP and NADPH (Wang et al., 2022). Furthermore, tolerant cultivars exhibited significantly higher ACP activity under LP (**Figure 5H**). Increased ACP activity promotes the hydrolysis of esterified P, releasing Pi into the cytosol and stabilizing the cytosolic phosphate pool (Plaxton and Tran, 2011). Adequate cytosolic Pi is required for triose phosphate export via the chloroplast triose phosphate translocator. By sustaining this counter-substrate supply, intracellular P recycling alleviates the feedback restriction on photosynthesis and supports continued Rubisco operation in the Calvin cycle (Rao and Terry, 1989; Kamoku et al., 2025). Overall, tolerant cultivars mitigate LP-induced photosynthetic inhibition through the coordinated regulation of stomatal function (facilitating P translocation) and biochemical maintenance. This integration ultimately supports a sustained advantage in biomass accumulation (Zhu et al., 2025).

### Redistribution of carbon and nitrogen metabolites and systemic root-shoot coordination

The synthesis and export of photosynthates represent a central physiological link between source leaves and sink organs. Under LP stress, the maintained SPS activity observed in tolerant genotypes acts as an important biochemical driver of source supply. As a rate-limiting enzyme in sucrose biosynthesis, the upregulation of SPS accelerates the conversion of triose phosphates into sucrose for long-distance export. Concurrently, this process releases Pi back into the chloroplast stroma, thereby helping to alleviate phosphate limitation in the Calvin cycle (Hashida et al., 2016; Stein and Granot, 2019). According to the source–sink relationship theory, this enhanced source supply is coupled with an increased sink demand from the roots. Tolerant genotypes exhibited relatively higher *I*_max_ and more extensive root proliferation, creating a substantial sink demand. This elevated root sink strength establishes a positive feedback loop that further stimulates SPS activity in the source leaves. In turn, this promotes targeted carbon allocation to the roots, supporting sustained root foraging. Through this coordinated source–sink regulation, the carbon assimilation advantage of tolerant genotypes is partially redirected toward root support rather than being confined to leaf metabolism (Yonekura et al., 2013; Thomas et al., 2022; Sun et al., 2025). Beyond metabolic flux, this root–shoot coordination operates within an integrated molecular regulatory network. Under P starvation, sucrose functions as a fundamental carbon skeleton and a key systemic signaling molecule. Accumulating evidence indicates that shoot-derived sucrose is transported via the phloem to the roots, where it interacts with the central PSR network. Specifically, sucrose signaling is required to trigger the expression of key phosphate transporters (e.g., *OsPTs*) and modulate the highly conserved PHR1-miR399-PHO2 signaling module that governs systemic Pi homeostasis (Bari et al., 2006; Lei et al., 2011).

Furthermore, LP treatment induced a broader reorganization of intracellular carbon and nitrogen metabolism. RF analysis identified FAA as a major predictor of low-P tolerance, with tolerant genotypes maintaining higher FAA and SSC levels. This finding highlights the multifaceted roles of FAA in LP. First, they function as compatible solutes for osmotic adjustment, thereby protecting cellular structures from stress (Živanović et al., 2020). Second, FAA serves as an internal buffering pool for nitrogen recycling, enabling flexible intracellular nitrogen reallocation without the need for energetically expensive *de novo* synthesis under ATP-limited conditions (Krouk and Kiba, 2020). Finally, they sustain metabolic homeostasis by acting as alternative substrates for the TCA cycle when carbohydrate metabolism is restricted (Batista–Silva et al., 2019). Consequently, the dynamic regulation of both SPS-mediated sucrose transport and the FAA pool supports the physiological plasticity required to cope with the metabolic stress imposed by LP stress.

RF and SEM analyses revealed a clear shift in the physiological determinants of biomass formation under LP treatment. Under P-sufficient conditions, biomass accumulation was primarily associated with photosynthetic carbon assimilation. However, under LP, statistical models indicated that reliance on photosynthesis alone was insufficient to predict growth. Instead, biomass formation showed strong associations with both carbon assimilation and carbon–nitrogen metabolism (**Figures 8A, B**). These results imply that low-P-tolerant genotypes depend not only on maintaining carbon supply but also on preserving metabolic homeostasis. Therefore, we propose that physiological adaptation to LP in rice involves an integrated strategy. This strategy is characterized by efficient root uptake, maintenance of photosynthetic function, and coordinated remodeling of carbon and nitrogen metabolism.

## Conclusion

This study demonstrated that LP-tolerant rice genotypes adapted to LP through an integrated strategy involving the optimization of root phosphorus uptake and coordinated adjustment of shoot metabolism. Under LP-related conditions, tolerant genotypes displayed a kinetic pattern combining higher uptake capacity with stronger affinity, which was associated with improved P acquisition. The preservation of photosynthetic performance was further supported by enhanced acid phosphatase activity and delayed leaf senescence. Moreover, the increased SPS activity promoted the preferential allocation of photosynthates to the roots, and the accumulation of free amino acids contributed to the re-establishment of the carbon–nitrogen metabolic balance. SEM further showed that under LP, tolerance depended not only on photosynthetic carbon assimilation but also on coordinated carbon–nitrogen metabolic regulation. Accordingly, root uptake kinetic traits and SPS activity may serve as promising physiological indicators of LP tolerance. Further validation across larger germplasm populations and under field conditions is required to determine their breeding utility for developing nutrient-efficient rice cultivars.

## Data Availability

The original contributions presented in the study are included in the article/**Supplementary Material**. Further inquiries can be directed to the corresponding authors.
