# Supplementary material for: Synergistic adaptation of rice root phosphorus uptake kinetics and leaf carbon–nitrogen metabolism under low-phosphorus conditions
Source: Front Plant Sci. 2026 May 18;17:1836788. doi: 10.3389/fpls.2026.1836788 (PMC13223106; doi:10.3389/fpls.2026.1836788)
Supplement: Supplementary file 1 [file DataSheet1.docx]

Supplementary Material

Table S1: Test rice germplasm.

| Variety  number | Germplasm | Variety  number | Germplasm | Variety  number | Germplasm | Variety  number | Germplasm |
| --- | --- | --- | --- | --- | --- | --- | --- |
| V1 | Q3-23 | V40 | HK 4617 | V79 | Y2-13 | V118 | ZKF 21-1 |
| V2 | Y3-37 | V41 | JND 771 | V80 | CJ 625 | V119 | YD 802 |
| V3 | Q5-18 | V42 | Y2-20 | V81 | Y3-19 | V120 | JJ 355 |
| V4 | Q3-17 | V43 | Q3-1 | V82 | Y3-11 | V121 | Q2-7 |
| V5 | C J677 | V44 | SL 23 | V83 | Q3-18 | V122 | Q5-12 |
| V6 | Q3-10 | V45 | SL 24 | V84 | Y3-17 | V123 | TH 2202 |
| V7 | Q3-16 | V46 | Q2-13 | V85 | JND 673 | V124 | Y2-22 |
| V8 | ZDS 809 | V47 | Q3-29 | V86 | Q3-22 | V125 | 22-2001 |
| V9 | Q5-6 | V48 | Q3-11 | V87 | Y3-39 | V126 | JH 808 |
| V10 | Q2-16 | V49 | HK 9877 | V88 | Q3-21 | V127 | DJ 701 |
| V11 | Y2-8 | V50 | Y3-12 | V89 | MYD 100 | V128 | Q3-20 |
| V12 | Y2-16 | V51 | JND 669 | V90 | Y2-21 | V129 | Y3-29 |
| V13 | LD 217 | V52 | DD 144 | V91 | JND 777 | V130 | DD 607 |
| V14 | Y3-26 | V53 | Y2-19 | V92 | JND 667 | V131 | JND 873 |
| V15 | TY 967 | V54 | Y3-33 | V93 | JD 937 | V132 | JD 722 |
| V16 | Y3-7 | V55 | Y3-38 | V94 | JD 155 | V133 | Q5-14 |
| V17 | Y3-14 | V56 | JG 981 | V95 | JJ 337 | V134 | Q2-17 |
| V18 | JYJ | V57 | Y3-24 | V96 | Q3-28 | V135 | Q5-8 |
| V19 | Y3-9 | V58 | T 35 | V97 | SJ 121 | V136 | JD 133 |
| V20 | 22-2008 | V59 | Y3-31 | V98 | Y3-41 | V137 | Y3-30 |
| V21 | DD 862 | V60 | Y3-32 | V99 | Y3-15 | V138 | Y2-17 |
| V22 | TH 2272 | V61 | JG 983 | V100 | HK 617 | V139 | DD 628 |
| V23 | Q2-8 | V62 | Y3-25 | V101 | Y3-10 | V140 | TH 2179 |
| V24 | Q3-30 | V63 | Q3-3 | V102 | Y3-3 | V141 | Q2-75 |
| V25 | Q2-1 | V64 | LD 20 | V103 | Y3-28 | V142 | HY 16 |
| V26 | Q2-3 | V65 | JD 36 | V104 | Q5-11 | V143 | JD 51 |
| V27 | Q3-13 | V66 | Y2-2 | V105 | TY 955 | V144 | Q3-4 |
| V28 | Q3-24 | V67 | Q3-25 | V106 | Y3-13 | V145 | HK 870 |
| V29 | Y3-42 | V68 | Q2-3 | V107 | Q3-37 | V146 | Q5-15 |
| V30 | Q3-15 | V69 | Y3-16 | V108 | Q3-27 | V147 | Y2-1 |
| V31 | Y3-34 | V70 | XN 968 | V109 | ZLJ 10 | V148 | Y2-23 |
| V32 | Q2-9 | V71 | Y3-35 | V110 | Q2-2 | V149 | Y2-5 |
| V33 | Q3-8 | V72 | ND 6118 | V111 | Q2-5 | V150 | JD 218 |
| V34 | Y3-7 | V73 | LD 1001 | V112 | Y2-11 | V151 | Q2-14 |
| V35 | Y2-3 | V74 | Y3-2 | V113 | Q3-12 | V152 | Y3-4 |
| V36 | Q5-17 | V75 | Y3-21 | V114 | TY 8704 | V153 | Y3-23 |
| V37 | Q2-10 | V76 | Q3-7 | V115 | Q3-9 | V154 | Y3-22 |
| V38 | DD 807 | V77 | Q5-9 | V116 | Q3-26 | V155 | Q5-16 |
| V39 | JIJ 338 | V78 | JND 705 | V117 | Q3-14 | V156 | LD 1136 |
| Note: CJ 667:Changjing 667; ZDS 809:Zhongduoshou 809; TY 967:Tongyu 967; JYJ: Jingyujing; DD 862:Dongdao 862; TH: Tonghe 2272; HK 4617:Hongke 4617; JND 771:Jing nongda 771; SL 23:Songliang 23; SL 24:Songliang 24; HK 9877:Hongke 9877; JND 669:Jinongda 669; DD 144:Dongdao 144; JG 981:Jingu 981; JG 983:Jingu 983; LD 20:Longdao 20; JD 36:Jiadao36; XN 968:Xinong 968; ND 6118:Nongda 6118; LD 1001:Longdao 1001;CJ 625:Chanjing 625; JND 673;Jinongda 673; MYD 1001:Muyudao 1001; JND 777;Jinongda 777; JND 667;Jinongda 667; JD 937:Jiudao 937; JD 155:Jida 155; JJ 337:Jijing 337; SJ 121:Suijing 121; HK 617:Hongke 617; TY 955;Tongyu 955; ZLJ 10:Zhonglongjing 10;TY 8704:Tongyu 8704. | | | | | | | |

Table S2 Composition of the modified Kimura B nutrient solution used in this study

| Reagent | Stock solution (mM) | Test concentration (mM) |
| --- | --- | --- |
| (NH_4_)_2_SO_4_ | 364.8 | 0.3648 |
| KH_2_PO_4_ | 182.2 | 0.1822 |
| KNO_3_ | 183 | 0.183 |
| K_2_SO_4_ | 91.2 | 0.0912 |
| Ca (NO_3_)_2_/Ca (NO_3_)_2_·4H_2_O | 365.0/349.7 | 0.3650/0.3497 |
| MgSO_4_/MgSO_4_·7H_2_O | 547.5/547.0 | 0.5475/0.5470 |
| H_3_BO_3_ | 30.1 | 0.0301 |
| CuSO_4_·5H_2_O | 0.3 | 0.0003 |
| ZnSO_4_·7H_2_O | 0.8 | 0.0008 |
| MnCl_2_·4H_2_O | 9.1 | 0.0091 |
| (NH_4_) _2_MoO_4_·4H_2_O | 0.5 | 0.0005 |
| Na_2_·EDTA | 22.2 | 0.0222 |
| FeSO_4_·7H_2_O | 20 | 0.02 |

Note: The Kimura B nutrient solution needs to be diluted 1000-fold when in use.

Table S3 Two-way ANOVA evaluating the main and interactive effects of phosphorus treatments (P) and cultivars (C) on plant growth and physiological traits at 0, 7, 14, and 21 days after treatment (DAT)

| Traits | DAT | Phosphorus (P) | Cultivar (C) | P × C Interaction |
| --- | --- | --- | --- | --- |
| Shoot dry weight | 0 | P = 0.876 | P < 0.01 | P =1.0 |
|  | 7 | P < 0.01 | P = 0.004 | P = 0.059 |
|  | 14 | P < 0.01 | P = 0.699 | P = 0.423 |
|  | 21 | P < 0.01 | P < 0.01 | P = 0.33 |
| Root dry weight | 0 | P = 0.878 | P < 0.01 | P = 1.0 |
|  | 7 | P < 0.01 | P = 0.055 | P = 0.158 |
|  | 14 | P < 0.01 | P < 0.01 | P < 0.01 |
|  | 21 | P < 0.01 | P < 0.01 | P < 0.01 |
| Root:Shoot Ratio | 0 | P = 0.876 | P < 0.01 | P = 1.0 |
|  | 7 | P = 0.799 | P = 0.004 | P = 0.006 |
|  | 14 | P = 0.83 | P < 0.01 | P < 0.01 |
|  | 21 | P = 0.148 | P < 0.01 | P < 0.01 |
| Phosphorus accumulation | 0 | P =0.879 | P =0.007 | P = 1.0 |
|  | 7 | P < 0.001 | P = 0.153 | P =0.412 |
|  | 14 | P <0.01 | P = 0.149 | P =0.229 |
|  | 21 | P <0.01 | P <0.01 | P = 0.019 |
| Chlorophyll a | 0 | P = 0.877 | P <0.01 | P = 1.0 |
|  | 7 | P < 0.001 | P = 0.002 | P = 0.296 |
|  | 14 | P < 0.001 | P < 0.001 | P = 0.009 |
|  | 21 | P < 0.001 | P < 0.001 | P = 0.001 |
| Chlorophyll b | 0 | P =0.876 | P = 0.007 | P = 1.0 |
|  | 7 | P < 0.001 | P = 0.222 | P = 0.682 |
|  | 14 | P < 0.001 | P < 0.001 | P = 0.707 |
|  | 21 | P < 0.001 | P < 0.001 | P = 0.089 |
| Total chlorophyll | 0 | P = 0.877 | P = 0.002 | P = 1.0 |
|  | 7 | P < 0.001 | P = 0.019 | P =0.407 |
|  | 14 | P <0.001 | P < 0.001 | P = 0.07 |
|  | 21 | P < 0.001 | P < 0.001 | P = 0.005 |
| Net photosynthetic rate | 0 | P = 0.877 | P = 0.013 | P = 1.0 |
|  | 7 | P < 0.001 | P = 0.023 | P = 0.02 |
|  | 14 | P < 0.001 | P < 0.001 | P < 0.001 |
|  | 21 | P < 0.001 | P < 0.001 | P < 0.001 |
| Stomatal conductance | 0 | P = 0.877 | P = 0.243 | P = 1.0 |
|  | 7 | P < 0.001 | P = 0.027 | P = 0.473 |
|  | 14 | P < 0.001 | P < 0.001 | P = 0.211 |
|  | 21 | P < 0.001 | P < 0.001 | P < 0.001 |
| Intercellular CO_2_ concentration | 0 | P = 0.877 | P = 0.149 | P = 1.0 |
|  | 7 | P < 0.001 | P = 0.916 | P = 0.716 |
|  | 14 | P < 0.001 | P = 0.049 | P = 0.675 |
|  | 21 | P < 0.001 | P < 0.001 | P = 0.147 |
| Transpiration rate | 0 | P = 0.877 | P = 0.174 | P = 1.0 |
|  | 7 | P < 0.001 | P = 0.783 | P = 0.463 |
|  | 14 | P < 0.001 | P = 0.007 | P =0.002 |
|  | 21 | P < 0.001 | P < 0.001 | P < 0.001 |
| Ribulose-1,5-bisphosphate carboxylase | 0 | P = 0.877 | P = 0.048 | P = 1.0 |
|  | 7 | P < 0.001 | P = 0.573 | P =0.037 |
|  | 14 | P < 0.001 | P < 0.001 | P = 0.007 |
|  | 21 | P < 0.001 | P < 0.001 | P < 0.001 |
| Sucrose phosphate synthase | 0 | P = 0.877 | P = 0.488 | P = 1.0 |
|  | 7 | P < 0.001 | P = 0.692 | P = 0.293 |
|  | 14 | P < 0.001 | P = 0.008 | P = 0.224 |
|  | 21 | P < 0.001 | P < 0.001 | P < 0.001 |
| Sucrose synthase | 0 | P = 0.877 | P = 0.002 | P = 1.0 |
|  | 7 | P < 0.001 | P = 0.012 | P = 0.612 |
|  | 14 | P < 0.001 | P = 0.004 | P = 0.489 |
|  | 21 | P < 0.001 | P = 0.011 | P =0.159 |
| Acid phosphatase | 0 | P = 0.877 | P < 0.001 | P = 1.0 |
|  | 7 | P < 0.001 | P < 0.001 | P = 0.017 |
|  | 14 | P < 0.001 | P < 0.001 | P = 0.001 |
|  | 21 | P < 0.001 | P < 0.001 | P < 0.001 |
| Soluble sugars | 0 | P = 0.877 | P < 0.001 | P = 1.0 |
|  | 7 | P < 0.001 | P = 0.009 | P = 0.02 |
|  | 14 | P < 0.001 | P < 0.001 | P < 0.001 |
|  | 21 | P < 0.001 | P < 0.001 | P < 0.001 |
| Soluble proteins | 0 | P = 1.0 | P < 0.001 | P = 1.0 |
|  | 7 | P < 0.001 | P < 0.001 | P = 0.812 |
|  | 14 | P < 0.001 | P < 0.001 | P = 0.275 |
|  | 21 | P < 0.001 | P < 0.001 | P = 0.004 |
| Free amino acids | 0 | P = 1.0 | P = 0.05 | P = 1.0 |
|  | 7 | P < 0.001 | P = 0.036 | P = 0.352 |
|  | 14 | P < 0.001 | P = 0.003 | P = 0.015 |
|  | 21 | P < 0.001 | P = 0.004 | P = 0.018 |


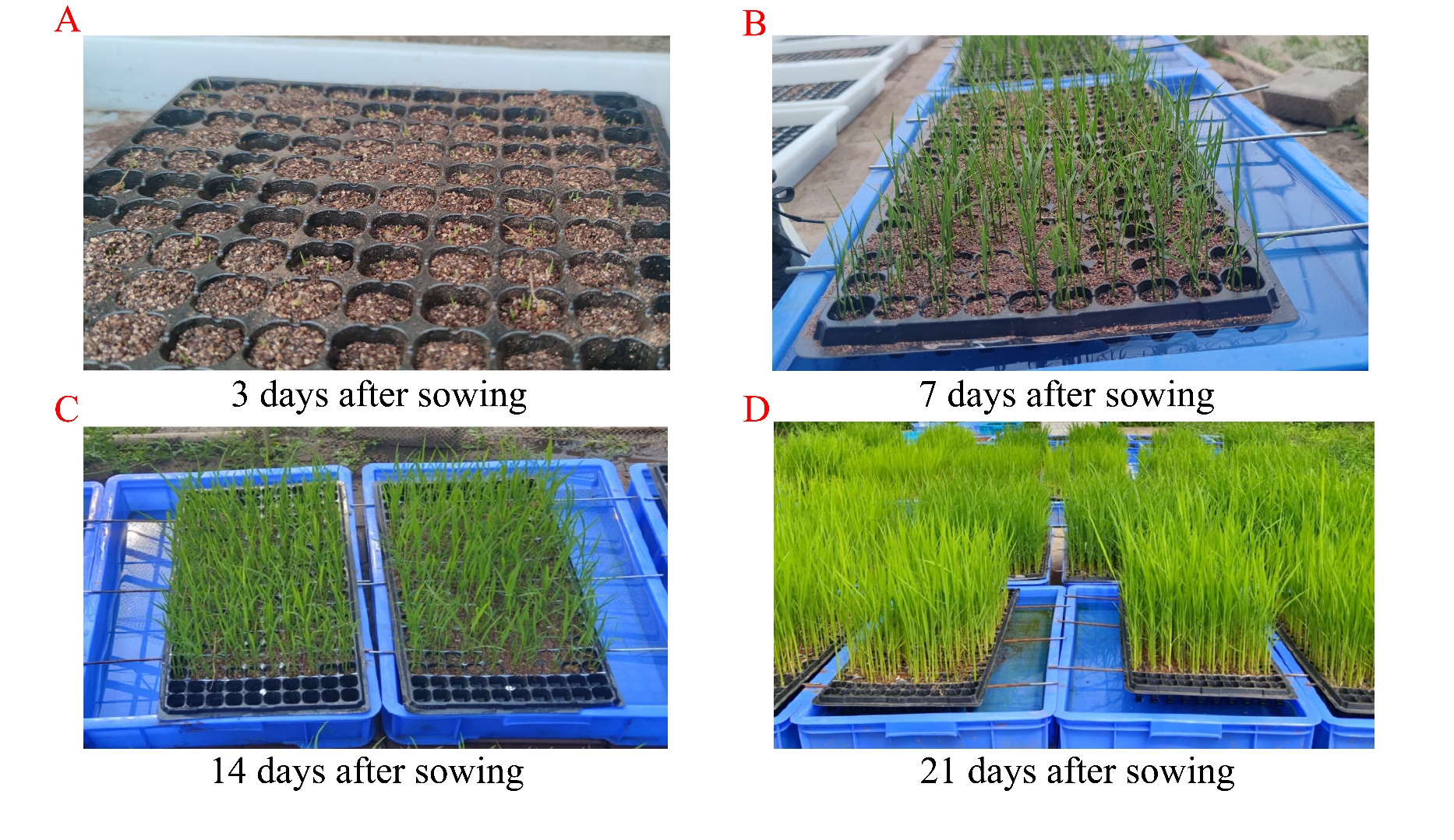


Figure S1. Sowing and Seedling Stage


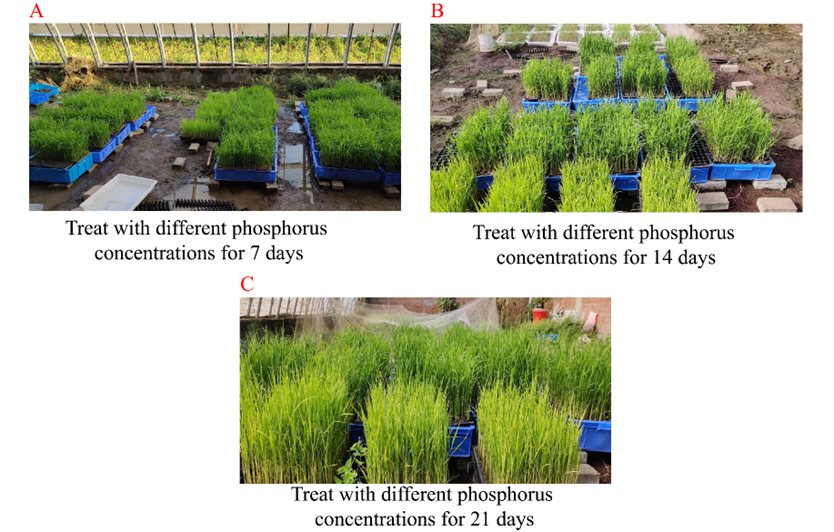


Figure S2. Different concentration treatments


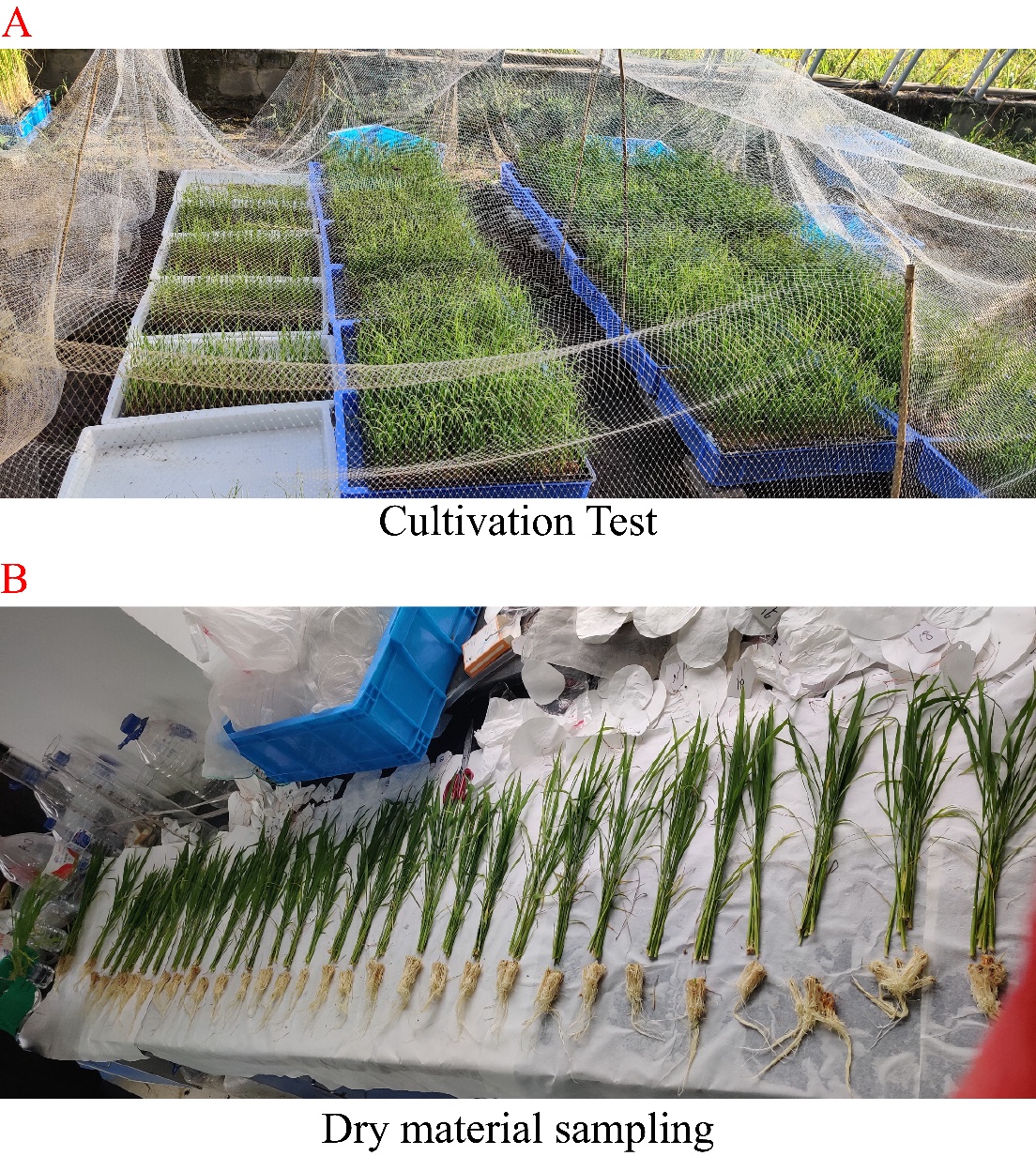


Figure S3. Culture and Sampling
